# Quality of Life of the Health Care Workers in the Pre-Retirement Period from the Private Sector of the Primary Health Care from the Skopje Region

**DOI:** 10.3889/oamjms.2015.097

**Published:** 2015-08-31

**Authors:** Iskra Gerazova Mujchin

**Affiliations:** Satcom, Skopje, Republic of Macedonia

**Keywords:** health care workers, pre-retirement period, private sector, public sector, quality of life, questionnaire

## Abstract

**BACKGROUND:**

The quality of life (QOL) of the workers in the pre-retirement period is an important line in their functioning, as well as in the process of their preparing for retirement.

**AIM:**

To assess the QOL of the health care workers - HCW (doctors and nurses/medical technicians) in the pre-retirement period from the private sector of the Primary Health Care (PHC).

**MATERIAL AND METHODS:**

We performed a cross-sectional, questionnaire-based study including 200 HCW in their pre-retirement period from the PHC from the Skopje region divided in two groups. The examined group (EG) included 100 HCW working in the private sector, whereas the control group (CG) consisted of 100 HCW employed in the public sector, matched to EG by age and duration of employment at the actual workplace. The QOL of the examinees was assessed by the World Health Organization Quality of Life - Bref questionnaire (WHOQOL - BREF).

**RESULTS:**

Examinees from both group assessed their QOL as good, i.e. there was no significant difference between the mean scores of EG and CG in regard to assessment of their QOL (3.7 vs. 3.6; p = 0.274). Regarding the satisfaction with their health, we found that examinees from EG are significantly more satisfied with their health than the examinees of CG as it was expressed by the obtained mean scores (3.9 vs. 3.6; p = 0.017). The mean scores of the domain assessing physical health and environment did not differ significantly between EG and CG (23.4 vs. 22.9; p = 0.187 and 25.7 vs. 24.9; p = 0.290, respectively). We found significant difference between EG and CG in regard to the mean scores assessing the psychological health (23.1 vs. 21.5; p = 0.003) and social life (11.6 vs. 10.1; p < 0.001).

**CONCLUSION:**

HCW from EG evaluated their QOL slightly better and they were more satisfied with their health than HCW from CG. In addition, HCW from EG assessed better their psychological health and social life than HCW from CG, whereas regarding the assessment of the physical health and environment, there was no difference between two groups.

## Introduction

The pre-retirement period is an important period of the people’s life as a precursor of the period that follows. The quality of life (QOL) of these people is an important line in their functioning, as well as in the process of their preparing for retirement.

Awareness of the need to assess the QOL of workers in the health sector is growing [1, 2]. Not just for themselves, but health-care staff provides higher quality services for their customers when they are healthy and have good quality of life [3]. Literature supports that high nursing workload adversely affects quality and safety of care and quality of working life [4, 5]. In addition, doctor’s discontent is a current topic for discussion in medical journals, and there seems to be a general agreement – at least within the profession itself – that job satisfaction among physicians is declining and it affects to the quality of working life [6]. The health care workers (HCW) are recognized as a vulnerable group of workers, due to their exposure to a number of hazards (physical, chemical, biological, psychosocial and ergonomic) at the workplace. Furthermore, HCW were selected as a priority for improvement of safety and health at work in the World Health Organization (WHO) Work Plan 2009-2012 (Priority 1.4) [7].

QOL is a broad-ranging concept, incorporating the person’s physical health, psychological state, level of independence, social relationships, personal beliefs and their relationship with salient features of the environment [8, 9]. QOL is defined as the reaction, either more cognitive or evaluative (life satisfaction) or affective (happiness, morale), to the congruence or discrepancy between a person’s standards, goals, values, and his/her actual situation, accomplishments, and so forth [10].

Broadly, subjective QOL involves the self-evaluation (expression of satisfaction or discontent, values and perceptions) of one’s personal circumstances in life [11]. Subjective wellbeing (positive and negative affect), happiness, life and needs satisfaction measures are often used as key indicators of subjective quality of life [12]. Subjective wellbeing is defined as the balance between positive and negative affect [13]. Life satisfaction is the cognitive evaluation of one’s life, which involves the comparison between one’s aspirations and achievements [11].

The QOL cannot be standardized, as it has different meanings for different individuals, depending on their objectives, goals and intentions [1, 14]. In addition, the QOL cannot be measured solely in terms of how long someone lives, because various factors can influence it, such as health, housing, work, leisure, and satisfaction, among other [14-17]. In the healthcare organizations, quality of work life (QWL) has been described as referring to the strengths and weaknesses in the total work environment [18, 19]. The WHO has defined QOL as “an individual’s perception of their position in life in the context of the culture and value systems in which they live and in relation to their goals, expectations, standards and concerns” [20, 21].

The individuals’ QOL is assessed by standardized questionnaires developed by different entities that are recognizing the multidimensional nature of this issue and emphasizing the personal perceptions of the QOL. One of the most used questionnaires for this purpose is the WHO QOL-BREF (WHOQOL-BREF) questionnaire which captures many subjective aspects of QOL [22-24].

The aim of the present study is to assess the QOL of the HCW in the pre-retirement period from the private sector of the Primary Health Care (PHC) from the Skopje region.

## Material and Methods

### Study design

A cross-sectional, questionnaire-based study was carried out in the period from November 2014 to April 2015 in close cooperation with the team from the Institute of Occupational Health of R. Macedonia, Skopje - WHO Collaborating Center. The QOL in the group of HCW in the pre-retirement period from the private sector of the PHC was compared with the QOL of the group of HCW employed in the public sector of the PHC.

### Study subjects

In the study were included 200 HCW of the PHC from the Skopje region divided in two groups.

The examined group (EG) consisted of 100 HCW (66 doctors and 34 nurses/technicians; 43 males and 57 females; mean age 59.5 ± 2.3 years, range 56 to 64 years) in their pre-retirement period (i.e. the last five years of their employment).

The control group (CG) included 100 HCW (51 doctors and 49 nurses/technicians; 35 males and 65 females; mean age 60.9 ± 1.9 years, range 56 to 65 years) employed in the public sector of the PHC, matched to EG by age and duration of employment at the actual workplace.

All study subjects provided written informed consent after a detailed explanation of the study and its purpose.

### Questionnaire

An interviewer-led questionnaire, i.e. the short version of the scale for assessing the QOL designed by the WHO experts team named WHOQOL - BREF (The World Health Organization Quality of Life - Bref) [25] was completed by all study subjects.

The questionnaire included 26 questions, two for the general QOL (question 1 - Q1) and overall health (question 2 - Q2) and 24 questions measuring four broad domains (physical health, psychological health, social relationships, and environment).

The physical health (Domain 1 - D1) is assessed by 7 questions about activities in daily living, dependence on medicinal substances and medical aids, energy and mobility, pain and discomfort, sufficient sleep and rest and good work capacity.

Assessment of the psychological health (Domain 2 - D2) includes questions about expressing of the someone’s feeling, i.e. experience of the body image and appearance, negative and positive feelings, self-respect, spirituality, religion, personal beliefs, thinking, learning, memory and concentration).

Domain for social relations (Domain 3 - D3) is assessed by the questions covering the area of experience in personal relationships, social support, and sexual activity.

Assessment of the environment area (Domain 4 - D4) includes questions covering the financial resources; freedom, physical security and safety; health and social care (accessibility and quality); home environment; opportunities for new information and skills obtaining; participation and opportunities for recreation/leisure; and physical environment (pollution, noise, traffic, climate, and transport).

In the scoring of the WHOQOL – BREF, the questions 1 and 2 are examined separately. Domain scores are scaled in a positive direction; higher scores denote higher quality of life, on a scale from 1 to 5 starting with *Poor QOL* to *Good QOL*. The main score of items within each domain is used to calculate the mean domain score. Calculation and transformation of the domain’s mean scores and interpretation of the obtained data is described elsewhere [8, 26].

### Limitations of the study

Limitations of the study included:

- Non-response bias -rejection of the examinees participating in the survey; and
- Reporting bias - unwillingness and indifference of examinees for giving truthful answers to the questions.

### Statistical analysis

Several statistical methods were used for the analysis of the data obtained:

- Analysis of the structure with measures of central tendency (mean, median and mode) and measures of statistical digression (standard deviation and standard error)
- Cronbach’s alpha coefficient for measuring of the internal group consistency;
- Identifying the factors of relations, proportions and ratios;
- Analysis of the relationship between individual statistical series with Mann-Whitney U- test and t-test for independent samples.

A *P*-value less than 0.05 was considered as statistically significant. Statistical analysis was performed using the Statistical Package for the Social Sciences (SPSS) version 11.0 for Windows.

## Results

The value of the Cronbach`s Alpha coefficient for the total scale was 0.91, indicating a good internal consistency of all questions in the questionnaire. Cronbach`s Alpha coefficient for the different domains was: D1 - 0821, D2- 0701, D 3 - 0.761 and D 4 - 0.809, indicating also a good internal consistency of all questions in each domain (Table 1).

**Table 1:** Cronbach`s Alpha coefficient

	Cronbach`s Alpha coefficient
D1 (physical health)	0.821
D2 (psychological health)	0.701
D3 (social relations)	0.761
D4 (environment)	0.809

As it is mentioned above, two questions from the WHOQOL-BREF questionnaire, i.e. “*How would you evaluate your quality of life?*” (Q1) and “*How satisfied are you with your health?*”(Q2), are examined separately, not as a part of the four domains mentioned above. The mean scores of the WHOQOL-BREF questionnaire are shown on Table 2.

**Table 2:** Display of the mean scores of the WHOQOL-BREF questionnaire

EG	Mean score	-95%CI	+95%CI	Minimum	Maximum	SD
**Q1**	3.7	3.57	3.88	2.0	5.0	0.780086
**Q2**	3.9	3.76	4.05	2.0	5.0	0.739847
**D1**	23.4	22.9	24.0	16.0	27.0	2.803965
**D2**	23.1	22.5	23.7	15.0	30.0	3.196889
**D3**	11.6	11.2	11.9	7.0	15.0	1.759563
**D4**	25.7	24.7	26.7	11.0	34.0	5.035390
**CG**
**Q1**	3.6	3.42	3.73	2.0	5.0	0.758776
**Q2**	3.6	3.47	3.78	2.0	5.0	0.760847
**D1**	22.9	22.2	23.5	15.0	27.0	3.267146
**D2**	21.5	20.7	22.3	12.0	30.0	4.100887
**D3**	10.1	9.7	10.5	5.0	13.0	2.029181
**D4**	24.9	23.9	26.0	10.0	36.0	5.366977

The mean scores of the Q1 (*How would you evaluate your quality of life?*) are similar for both groups, i.e. according to the result of the Mann-Whitney U test. The difference registered among the two groups is statistically non-significant (3.7 *vs*. 3.6; P = 0.274) (Table 3).

**Table 3:** Difference between EG and CG in regard to the first two questions

	Rank Sum	Rank Sum	U	Z	P-level
**Q1**	9264.000	10239.00	4413.000	-1.09472	0.274
**Q2**	9077.000	11023.00	4027.000	-2.37742	0.017

According to Mann-Whitney U-test for the Q2 (*How satisfied are you with your health?*). The difference registered between the two groups is statistically significant (P = 0.017) (Table 3).

Regarding the question Q1, the examinees from EG (44.0%) assessed their QOL as good. 35.0% of the examinees stated their QOL as neither good nor bad. 16.0% believe that their QOL is very good and only 3.0% assessed it as bad. Two participants didn`t answer the question (Table 4).

**Table 4:** Evaluation of their quality of life (Q1) by study subjects

Answer	EG		CG	
	Number	%	Number	%
Dissatisfied	3	3.0	7	7.0
Neither satisfied. nor dissatisfied	35	35.0	36	36.0
Satisfied	44	44.0	46	46.0
Very satisfied	16	16.0	9	9.0
Total	98	98.0	98	98.0

Almost half of examinees from the CG (46.0%) assessed their QOL as good. 36.0% of the examinees stated their QOL as neither good nor bad. 9.0% of the examinees assessed their QOL as very good and only 7.0% assessed it as bad. Two participants did not answer the Q1 (Table 4).

Regarding the Q2, 60.0% of EG was satisfied with their health. 18.0% were very satisfied. 17.0% were neither satisfied nor were dissatisfied and only 5.0% dissatisfied with their health (Table 5).

**Table 5:** Evaluation of satisfaction with their health (Q2) by study subjects

Answer	EG		CG	
	Number	%	Number	%
Dissatisfied	5	5.0	9	9.0
Neither satisfied. nor dissatisfied	17	17.0	27	27.0
Satisfied	60	60.0	56	56.0
Very satisfied	18	18.0	8	8.0
Total	100	100.0	100	100.0

Regarding the examinees from the CG, almost half of them (56.0%) were satisfied with their health. 27.0% were neither satisfied nor dissatisfied. 9.0% were dissatisfied and 8.0% were very satisfied with their health (Table 5).

Regarding the domain`s results, Table 6 gives the difference between EG and CG obtained by testing with t-test for independent samples.

**Table 6:** Difference between EG and CG in regard to the four domains

Domain	t- value	P-value
**D1**	-1.32392	0.187
**D2**	-3.03861	0.003
**D3**	-5.62212	0.000
**D4**	-1.05988	0.290

According to the results of the t-test, the difference registered between the two groups in the terms of the D1 was statistically non-significant (Table 6). The mean score of the examinees from EG in terms of the D1 was higher (23.4 ± 2.8) suggesting that under D1 the examinees where “generally well satisfied”. The mean score of D1 for CG was 22.9 ± 3.3 suggesting that under D1 examinees were also “generally well satisfied “with their physical health (Table 2).

The mean score of the D2 registered in EG (23.1 ± 3.2) was significantly higher than its value registered in CG (21.5 ± 4.1) (P = 0.003) (Table 6). It suggested that under D2 the examinees from EG are “generally satisfied”, whereas the examinees from CG are “moderately satisfied” with their psychological health.

The mean scores of the D3 in EG and CG were 11.6 ± 1.6 and 10.1 ± 2.0, respectively (Table 2). The difference between its vales was statistically significant (P = 0.000) (Table 6). This finding indicated that the examinees from EG are generally “satisfied” and the examinees from CG are “neither satisfied nor dissatisfied” with their social life.

The mean scores of the D4 in EG and CG were 25.7 ± 5.0 and 24.9 ± 5.4, respectively. The difference between mean scores was statistically non-significant (P = 0.290) (Table 6). Under the D4 examinees from both groups were “moderately generally and very satisfied” with their environment area.

## Discussion

The Macedonian health care system consisted of three pillars i.e., of primary, secondary and tertiary health care. The PHC including private and public sector is the first line of the national health system. In the present study we assessed the QOL of the HCW from the PHC in their pre-retirement period, i.e. in the last five years of their employment using the WHOQOL – BREF questionnaire. To our knowledge, in the existing literature there is no study on QOL in HCW from primary care settings.

The study subjects from both groups evaluated their QOL as good, i.e., there was no significant difference between the two groups in regard to the evaluation of their QOL (Q1). In addition, we registered significant difference between groups regarding the satisfaction with their health (Q2). Namely, according to their answers to Q2, the examinees from EG (i.e. primary HCW from the private health sector) are more satisfied with their health than the primary HCW from the public sector. The difference could be explained by the differences between the work in private and public sector (i.e. better work conditions. better valorization of the work efforts. and higher salaries in the private sector) that is very important for each individual and also can influence the perception for their QOL and health condition.

The mean scores obtained for D1 did not differ significantly, indicating that the examinees of both groups are generally satisfied with their physical health (i.e. activities in daily living, dependence on medicinal substances and medical aids, energy and mobility, pain and discomfort, sufficient sleep and rest and good work capacity). Somewhat different results regarding D1 were obtained in the study conducted by Barrientos et al. [27] on Chileans hospital nurses. Majority of them assessed their physical health as bad and the authors explained it by multiple roles of the examinees (wife, mother, housewife, nurse, etc.) that led to feeling of overload.

The second domain (D2) of the WHOQOL – BREF covers the psychological health of the examinees including how noticeable is sense-experience for persons image and appearance, negative and positive feelings, self-respect, spirituality, religion, personal beliefs, thinking, learning, memory, and concentration. According to the mean score, the examinees from the EG generally are “generally satisfied”, whereas the examinees from the CG are “moderately satisfied”. HCW from the private sector are more satisfied with their psychological health than the public HCW and the registered difference is statistically significant. Regarding the psychological aspects, some authors conclude that, in female HCW, the work demands could suppose a lack of attention for family responsibilities, entailing repercussions for their private life that could reflect in feelings of guilt, which are assessed within this domain [28]. The literature review reveals that the stress at the workplace in nurses can contribute to poor job satisfaction, poor patient outcome, and poor perception of psychological and physical health and in extreme cases, suicide [29]. A cross-sectional Danish study used and effort – reward model to test the association with psychological health and poor self-rated health. Nurses were reporting poor health, as study results demonstrated statistical significance for overall poor general health, poor psychological wellbeing, gastrointestinal complaints, cardiovascular complaints, and musculoskeletal complaints [30]. In other study on staff in military hospitals in Paris and its urban area, psychosocial risk factors were found to be associated with moderate or poor perceived health. Among work characteristics, two variables were associated with moderate or poor perceived health, the ergonomic score and the occupational profile [31].

Modifying health behaviors and establishing social networks are considered as key elements in improving a person’s perception of QOL [29, 32]. For some researchers, social interaction is inherent to women’s nature and an interpersonal support obtained important scores in the perception of women’s health state [27]. Other authors has noticed that adverse social relationships and job characteristics are associated with poor health [33-36]. According to the mean score of the D3 (that covers the area of experience in personal relationships, social support and sexual activity), the examinees from EG are generally “satisfied”, whereas the examinees from CG are generally “neither satisfied nor dissatisfied”. The mean score of the examinees from EG is significantly higher than its value obtained from the examinees from the CG.

D4 covers the environment area (financial resources; freedom, physical security and safety; health and social care: accessibility and quality, home environment; opportunities for new information and skills obtaining; participation and opportunities for recreation/leisure; physical environment: pollution, noise, traffic, climate and transport). According to the mean scores of the D4, they are “moderately, generally and very satisfied.” Similarly to our findings, results from other studies indicated that older age had a positive impact on the physical and environmental domains within the QOL assessment in HCW [2, 37]. Furthermore, findings from several studies indicate better QOL scores in the domains of mental and physical health dimensions in older examinees, suggesting their better mental and physical health compared to younger examinees [27, 28, 38, 39].

Looking in general, an interesting view regarding the QOL in the European countries is showed in the Survey of Health, Ageing and Retirement in Europe. Namely, according to CASP (C-control, A-autonomy, S- self-realization, P-pleasure) - 12 scale values from 12 to 48, the scores are subsequently classified into four levels of the QOL. 39-41 indicating very high QOL, 37-39 high QOL, 35-37 moderate QOL, and values below 35 low QOL. Findings of the study suggested that the QOL is relatively low in Greece (33.43), Italy (34.26), and Spain (35.20) and relatively high in Switzerland (40.47), the Netherlands (39.07) and Denmark (39.76). Thus, there is evidence of a North-South gradient in degree of the QOL across the European countries. These findings indicated that differences in the QOL between younger and older age groups (lower QOL with older age) are relatively large in southern European countries; as well as that the QOL is consistently associated with socio-economic status (educational degree and income) and with the health indicators (better health with better QOL) in all European countries included in the study [40].

In conclusion, in a cross-sectional, questionnaire-based study on QOL of the HCW in the pre-retirement period from the private sector in the PHC from the Skopje region we found that these workers evaluated their QOL slightly better and that they were more satisfied with their health than the HCW in the pre-retirement period from the public sector of the PHC. In addition, HCW from private sector assessed better their psychological health and social life than HCW from public sector, whereas regarding the assessment of the physical health and environment there was no difference between two groups. Our findings indicate that health policy makers should work on programs that will maintain and improve the QOL in order to enable better preparing for retirement of these workers.
